# ^18^F-FDG PET/CT代谢参数在非小细胞肺癌预后评估中的应用及进展

**DOI:** 10.3779/j.issn.1009-3419.2019.03.09

**Published:** 2019-03-20

**Authors:** 涛 王, 振光 王

**Affiliations:** 266100 青岛，青岛大学附属医院核医学科 Department of Nuclear Medicine, the Affiliated Hospital of Qingdao University, Qingdao 266100, China

**Keywords:** 肺肿瘤, 肿瘤代谢参数, 预后评估, Lung neoplasms, Metabolic parameters, Prognostic evaluation

## Abstract

基于肺癌肿瘤-淋巴结-转移（tumor-node-metastasis, TNM）分期的治疗方案制定和预后评价是目前国内外肺癌指南中的基本原则。18氟代脱氧葡萄糖正电子发射计算机断层显像（^18^F-deoxyglucose positron emission tomography/computed tomography, ^18^F-FDG PET/CT）代谢参数如标准摄取值（standardized uptake value, SUV）、肿瘤代谢体积（metabolic tumor volume, MTV）、病灶糖酵解总量（total lesion glycolysis, TLG）可以反映肿瘤侵袭性的信息，提供额外的预后信息。将量化的肿瘤代谢负荷MTV、TLG联合传统的TNM分期对患者进行危险分层，作为一种新的分期方式可以辅助临床医师制定更为合适的治疗方案。^18^F-FDG PET/CT图像纹理分析作为一种新兴研究方法，可以量化肿瘤内放射性摄取的空间分布异质性，进而了解肿瘤的生物学特征。本文对^18^F-FDG PET/CT代谢参数在非小细胞肺癌患者预后评估的应用进行阐述。

## Abstract (en)

Tumor-node-metastasis (TNM) staging system is the most important basis for making therapeutic decisions and predicting prognosis of lung cancer patients. The metabolic parameters including standardized uptake value (SUV), metabolic tumor volume (MTV) and total lesion glycolysis (TLG) measured by ^18^F-fluorodeoxyglucose-positron emission tomography (^18^F-FDG PET/CT) associate with tumor aggressiveness and can provide additional prognostic information. A new staging methodology combines the conventional cTNM with the metabolic tumor burden quantified from the PET images is a better predictor of overall survival with superior stratifying power may help oncologists to make better treatment plans. ^18^F-FDG PET/CT image texture analysis, as an emerging research tool, is used to quantify the spatial heterogeneity of radioactive uptake in tumors, thereby to explore the biological characteristics of the tumor. This article reviews developments in evaluating the ^18^F-FDG PET/CT metabolic parameters and its role as a prognostic factor for non-small cell lung cancer.

肺癌是世界上发病率最高的恶性肿瘤，其中非小细胞癌（non-small cell lung cancer, NSCLC）占肺癌的80%-85%^[1]^。准确的分期有助于制定合适的治疗方案和判断预后，目前国际上最新的分期标准是国际抗癌联盟（Union for International Cancer Control, UICC）以及美国癌症联合会（American Joint Committee on Cancer, AJCC）联合发布的第8版的肿瘤-淋巴结-转移（tumor-node-metastasis, TNM）分期^[2]^。即使在同一分期的患者，由于个体异质性的存在，患者的预后也不尽相同，例如性别、年龄、肿瘤病理学类型及病人体力状态、体质量下降等因素对预后都有潜在的影响^[3, 4]^。正电子发射型计算机断层显像（positron emission computed tomography, PET）可以提供半定量代谢参数和定量参数，例如标准摄取值（standardized uptakevalue, SUV）、肿瘤代谢体积（metabolic tumor volume, MTV）和病灶糖酵解总量（total lesion glycolysis, TLG）。MTV和TLG在多种肿瘤中被证实是独立于TNM分期的预后影响因子，如鼻咽癌、食管癌、胰腺癌、卵巢癌、淋巴瘤^[5-9]^等。因此，肿瘤体积或负荷可能是一个潜在的新的预后因素，可以在TNM分期的基础上进一步进行风险分层。本文将从肿瘤代谢参数在NSCLC预后评估的应用及发展趋势进行阐述。

## 1 SUVmax在NSCLC预后评估中的应用

SUV半定量代谢指标，是目前临床最常用的指标，SUV基本定义为局部组织摄取的显像剂的放射性活度与全身平均注射活度的比值。常用的指标有最大标准摄取值（SUVmax）与平均标准摄取值（SUVmean）。SUVmax为感兴趣区内摄取FDG最大的体素，SUVmean为感兴趣区所有体素平均摄取值。肿瘤组织对^18^F-FDG摄取反映了肿瘤的代谢活性，是肿瘤的一个重要的生物学特性。SUV值的高低与同一病理类型肿瘤细胞分化程度有关。一般来说，肿瘤恶性程度越高，SUV值也越高。原发肿瘤的SUVmax对初诊NSCLC患者有很好的预测作用。

Takeda等^[10]^对283例行立体定向放疗（stereotactic body radiation therapy, SBRT）的早期NSCLC患者（T1a-2N0M0）分析，原发灶SUVmax越高，患者总体生存率（overall survival, OS）及无病生存期（disease-free survival, DFS）越差。Ren等^[11]^对182例肺鳞癌患者术后预后研究显示，原发灶SUVmax以13.0为界分为两组，SUVmax > 13.0组与SUVmax≤13.0组患者的中位总生存期分别为56个月和87个月，差异具有统计学意义。一项纳入36项研究^[12]^的*meta*分析显示，术前原发灶SUVmax越高，预后越差。虽然部分研究显示SUVmax是NSCLC预后影响因素，但这一结论仍受到争议。首先，SUV值会受到许多因素的影响，如病人体质量、血糖水平、注射放射活度、图像重建参数、分辨率、ROI定义参数^[14]^等。其次，SUV没有考虑肿瘤体积对预后的影响，仅反映病灶单一体素的代谢水平。Choi等^[15]^研究发现葡萄糖转运载体Glut-1、Glut-3在肿瘤组织的高表达与FDG摄取存在正相关。但是Wang等^[16]^在肺炎FDG摄取与Glut-1、Glut-3表达的相关性研究中发现，Glut-1、Glut-3也可在炎性细胞如淋巴细胞、单核细胞、巨噬细胞高表达，同样表现出FDG高摄取，所以肿瘤局部SUVmax可能存在炎性细胞高摄取的干扰，导致SUVmax不能准确代表肿瘤的代谢活性。

Hoang等^[17]^对214例未行手术治疗的Ⅲ期和Ⅳ期NSCLC分析，以原发灶中位SUVmax以11.1为界，SUVmax < 11.1组和SUVmax≥11.1组中位总生存期分别为16个月和12个月，两组之间无统计学差异，单因素和多因素分析显示SUVmax均不是预后因素。Burdick等^[18]^对72例行SBRT的早期（T1-2N0M0）NSCLC分析发现，原发灶SUVmax均不是远处转移或者OS的预后因子。Agarwal等^[19]^对早期（Ⅰ期和Ⅱ期）可行手术治疗的363例NSCLC进行研究，以原发灶最佳临界值SUVmax 8.2二分法对所有患者分组，单因素和多因素分析显示，SUVmax均没有很好的预后作用。

## 2 肿瘤代谢负荷在NSCLC预后评估的应用

MTV和TLG包含了肿瘤体积和代谢两方面信息，较SUV能更全面地反映肿瘤负荷。MTV为肿瘤代谢体积，代表肿瘤组织有较高代谢活性的体积。最常用的测量方法为固定阈值法，通过设定阈值，软件对区域内病灶进行容积分割，勾画出感兴趣区自动计算肿瘤体积。相对于人工勾画肿瘤边缘，此方法更加简便易行，耗时少，可重复性高。目前MTV常用的SUV阈值有SUV=2.5^[20]^，40%-70%SUVmax^[21]^，临床上最常应用SUV=2.5计算MTV。TLG为MTV与SUVmean的乘积，既包括肿瘤代谢活性，也能反映肿瘤代谢体积。全身病灶总糖酵解值（whole-body total lesion glycolysis, TLGwb）和全身肿瘤代谢体积（whole-body metabolic tumor volume, MTVwb）分别为全身病灶TLG、MTV之和，反映全身肿瘤代谢负荷。

Lee等^[22]^首先通过MTV对NSCLC进行预后评估，并对19例（18例NSCLC，1例小细胞肺癌）肺癌患者（Ⅰ期-Ⅳ期）进行分析，患者中位生存期为14.8个月，结果发现原发灶的MTV每增加25 mL，疾病进展和死亡的风险增加2.8倍，而原发灶的SUV并不具有预后价值。该作者另一项研究^[23]^纳入61例Ⅰ期-Ⅲ期NSCLC患者，以原发灶MTV中位数13.6 mL分组，MTV≤13.6组与MTV > 13.6组中位生存期分别为41.9个月和18.9个月。两组无疾病进展率分别为60%、39.7%，两组之间存在显著差异。MTV每升高41 mL，死亡风险增加53%，更好地证实了MTV的预后作用。

Satoh等^[24]^对88例行SBRT的Ⅰ期NSCLC患者进行分析，对于肿瘤直径 > 3 cm的肺癌患者，原发灶的MTV和TLG对DFS有预测作用。梁萌等^[25]^对放疗前行PET/CT检查的170例Ⅰ期-Ⅲ期NSCLC分析，结果显示1年、3年、5年OS率分别为82.4%、43.3%、24.7%。单因素生存分析示年龄、N分期、cTNM分期、肿瘤大小、原发灶的SUVmax、MTV及TLG为预后影响因素。多因素分析显示仅有年龄、原发灶MTV为总生存期预后影响因素。

Hyun等^[26]^回顾性纳入194例Ⅲa期行手术治疗的NSCLC患者，115例非手术治疗的（Ⅲa期50例，Ⅲb期65例）NSCLC患者。在手术组，在校正年龄、性别、组织病理学、TNM分期及术后治疗方案后，原发灶的MTV、TLG是OS的独立预后因素，MTV每升高1倍，死亡风险增加1.22倍。在非手术治疗组，MTV、TLG均不能预测预后，可能是由于非手术治疗患者总体预后较差。原发灶SUVmax在手术组和非手术组均不能预测预后。Sharma等^[27]^分析60例Ⅲa期-Ⅳ期NSCLC患者资料，患者均接受以铂类药物为基础的化疗，结果显示原发灶的基线MTV和TLG均为OS的预后因素，而原发灶的基线SUVmax没有预后作用。Salavati等^[28]^也证实了原发灶的MTV、TLG是比SUVmax更好的预后因子。

## 3 全身肿瘤代谢负荷在NSCLC预后评估中的应用

肿瘤是全身系统的疾病，进展到晚期往往发生转移，在预后评估中需要考虑原发灶以外的转移灶对其产生的影响。近年来更多学者对全身肿瘤代谢负荷做了深入的研究。全身肿瘤代谢负荷包括了原发灶、转移淋巴结及远处转移灶的代谢负荷，更全面反映了全身代谢信息。

Liao等^[29]^回顾性纳入169例非手术Ⅰ期-Ⅳ期NSCLC患者，结果显示MTVwb、TLGwb、肿瘤原发灶、转移淋巴结、远处转移灶的MTV和TLG均是独立于TNM分期的预后因素，且均优于原发灶SUVmax和SUVmean。该学者^[30]^另一项针对92例Ⅳ期NSCLC患者的研究，同样也证实MTVwb与TLGwb是独立于TNM分期的独立预后因素，且均优于SUVmax和SUVmean。Zhang等^[31]^对术前行PET/CT检查的104例Ⅰ期-Ⅳ期NSCLC患者进行分析，高MTVwb组（≥37.34），高TLGwb（≥407.48）组OS明显低于低MTV组（< 37.34）和低TLG（< 407.48）组。MTVwb和TLGwb均为独立于TNM分期、手术方式、化疗方案的预后影响因素，而原发灶的SUVmax、SUVmean没有很好的预测作用。

Zhang等^[32]^对术后行PET/CT检查的142例NSCLC病人进行生存分析。与术后FDG-PET/CT表现为阴性的病人相比，肿瘤FDG摄取的病人的生存率明显降低。对于肿瘤FDG浓聚的病人，分别以MTVwb、TLGwb、SUVwbmax中位数为临界值，采用二分法进行分组，组间OS有显著差异。在校正了病人年龄、性别、TNM再分期等因素后发现，MTVwb和TLGwb均为OS的预后因素。

目前TNM分期依旧是临床应用最广泛的分期方法，近年来有学者提出将传统的TNM分期与肿瘤代谢负荷结合，创造出新的分期算法。Zhang等^[33]^首先提出TNM分期联合MTVwb的预后指标，称之为PET/CT体积预后指数（PVP index），PVP指数=0.360In（MTVwb）+0.424I（TNM=Ⅲ）+0.890I（TNM=Ⅳ），I代表 1，当患者的TNM分期与括号中分期一致时，相反为0。他们对328例NSCLC进行生存分析，多因素分析显示PVP的分层能力高于TNM分期，高PVP指数的患者预后更差，PVP是更优的分层方法。Lapa等^[34]^同样也提出了临床TNM分期和PET/CT量化的全身肿瘤活性代谢体积MTVwb联合的分期算法，命名为TNM-PET（cTNM-P）分期。传统的TNM分期通过MATV-WB临界值对每一期再进行分层，通过这种方法，形成了新的分期系统，在每个亚组，MATV-WB≥截止点，患者生存率更低。与常规TNM分期相比，cTNM-P分期呈现更高的预测能力。该分期系统可能有助于鉴别预后更差和更高的复发风险和死亡率的NSCLC患者。

Finkle等^[35]^回顾性纳入330例Ⅱb期、Ⅲa期、Ⅲb期的NSCLC，并通过MTVwb对Ⅲa期NSCLC进行组内分层，以MTV最佳临界值29.2 mL分层，定义低MTV组为Ⅲa期，高MTV组为Ⅲa+期，两组间OS有差异（*P* < 0.01），中位生存期分别为2.92年、1.47年。而Ⅲa-期与Ⅱb期组间OS无明显差异（*P*=0.485），Ⅲa+期与Ⅲb期OS组间亦无差异（*P*=0.459）。因此对于同组内异质性高的Ⅲa期的患者，利用MTV进行危险分层，对于低危险组可以行手术治疗，高分险组更适合化疗和生物治疗。

上述研究没有否认TNM分期的价值，而是在此基础上将量化的肿瘤代谢负荷与其联合，并对患者进行危险分层。这种对患者进行量化评估的方式也有利于精准医疗和个体化治疗的发展推进。

## 4 PET/CT代谢参数在NSCLC疗效评估中的应用

PET/CT代谢参数指标治疗前后的变化率能较准确地反映肿瘤疗效。Moon等^[36]^回顾性纳入52例高分化NSCLC患者，1个周期化疗后TLG变化率≥50%的患者PFS更高，因此TLG变化率可以作为NSCLC化疗后的评价指标。陈美洁等^[37]^回顾性分析了18例Ⅲ期-Ⅳ期NSCLC患者，均接受以铂类为基础的一线化疗方案。通过ROC曲线分析得出以30%SUVmax勾画，MTV和TLG的变化率分别为56.3%、62.2%时可以判别出化疗敏感者，而基线状态下的上述指标与化疗敏感性的预测关系不明确。

纳武单抗是一种抗程序性死亡受体1（programmed cell death protein 1, PD-1）抗体，用于既往治疗过的NSCLC患者。一项初步研究^[38]^探讨了^18^F-FDG PET/CT预测纳武单抗早期阶段治疗反应的可行性。研究纳入24例NSCLC患者，计算治疗前后SUVmax、MTV、TLG，并行PD-L1表达和肿瘤浸润淋巴细胞的免疫组织化学分析。基于RECIST1.1，29%的患者达部分缓解（partial response, PR），SUVmax、MTV和TLG对应数据分别为29%、25%和33%。PET/CT对纳武单抗治疗1个月后PR（100% *vs* 29%）和疾病进展（100% *vs* 22.2%）的预测率高于CT。多因素分析显示纳武单抗治疗后的^18^F-FDG摄取是独立预后因素。

## 5 ^18^F-FDG PET/CT图像纹理分析在NSCLC预后评估中的应用

MTV与TLG仅仅反映了总体上肿瘤的代谢状态，^18^F-FDG PET/CT图像纹理分析作为一种新兴研究方法，可以量化肿瘤内放射性摄取的空间分布异质性，进而了解肿瘤的生物学特征。Cook等^[39]^对53例行放化疗NSCLC患者的研究中发现，图像纹理参数糙度与患者的PFS、局部无进展生存（local PFS, LPFS）和OS相关，且为三者的预后因子；粗糙度高患者死亡危险度较粗糙度低者高约5倍。参数对比度和忙度与患者的PFS和LPFS相关。Ha等^[40]^对30例NSCLC患者（腺癌17例，鳞癌13例）PET图像进行分析，获取PET图像的SUVmax和24个纹理参数，发现SUVmax和15个纹理参数在肺腺癌和鳞癌之间差异有统计学意义，进而说明NSCLC不同病理类型之间PET图像的瘤内代谢异质性可能存在差异。随着肿瘤精准治疗和临床大数据的逐渐推广应用，基于机器学习的FDG PET显像纹理分析将具有广阔的临床应用前景。

近年来，用于评估恶性肿瘤增殖活性的显像剂^18^F-脱氧胸腺嘧啶核苷（fluorothymidine, FLT）和评估肿瘤内乏氧状态的显像剂^18^F-米索硝唑（fluoromisonidazole, FMISO）在NSCLC预后评估中也有较多的应用^[41, 42]^，在未来可以更为广泛地应用于临床。

综上所述，肿瘤代谢负荷MTV、TLG相对于单一体素的SUVmax，均是在3D维度测量肿瘤体积及代谢活度，可以提供更丰富的肿瘤侵袭性的信息，是比原发性肿瘤SUVmax更好的预后因子，是独立于肿瘤分期和其他临床因素的预后影响因素。肿瘤全身代谢负荷MTVwb、TLGwb将全身病灶的代谢信息进行整合，更能体现肿瘤的侵袭性，具有更优的预后价值。将量化的肿瘤代负荷MTV、TLG联合传统的TNM分期对患者进行分层，辅助临床医师制定更为合适的治疗方案。图像纹理分析的应用日益广泛，可以作为传统代谢参数的补充，提供更全面的预后信息，具有很好的应用前景。
